# Role of the Gut Microbiome in Beta Cell and Adipose Tissue Crosstalk: A Review

**DOI:** 10.3389/fendo.2022.869951

**Published:** 2022-05-12

**Authors:** José Ignacio Martínez-Montoro, Miguel Damas-Fuentes, José Carlos Fernández-García, Francisco J. Tinahones

**Affiliations:** ^1^ Department of Endocrinology and Nutrition, Virgen de la Victoria University Hospital, Instituto de Investigación Biomédica de Málaga (IBIMA), Faculty of Medicine, University of Málaga, Málaga, Spain; ^2^ Centro de Investigación Biomédica en Red-Fisiopatología de la Obesidad y Nutrición (CIBERObn), Instituto de Salud Carlos III, Madrid, Spain; ^3^ Department of Endocrinology and Nutrition, Regional University Hospital of Málaga, Instituto de Investigación Biomédica de Málaga (IBIMA), Faculty of Medicine, University of Málaga, Málaga, Spain

**Keywords:** adipose tissue, beta cell, insulin resistance, diabetes, adipokines, gut microbiota, inflammation

## Abstract

In the last decades, obesity has reached epidemic proportions worldwide. Obesity is a chronic disease associated with a wide range of comorbidities, including insulin resistance and type 2 diabetes mellitus (T2D), which results in significant burden of disease and major consequences on health care systems. Of note, intricate interactions, including different signaling pathways, are necessary for the establishment and progression of these two closely related conditions. Altered cell-to-cell communication among the different players implicated in this equation leads to the perpetuation of a vicious circle associated with an increased risk for the development of obesity-related complications, such as T2D, which in turn contributes to the development of cardiovascular disease. In this regard, the dialogue between the adipocyte and pancreatic beta cells has been extensively studied, although some connections are yet to be fully elucidated. In this review, we explore the potential pathological mechanisms linking adipocyte dysfunction and pancreatic beta cell impairment/insulin resistance. In addition, we evaluate the role of emerging actors, such as the gut microbiome, in this complex crosstalk.

## 1 Introduction

The global prevalence of overweight and obesity has dramatically increased in the last few decades with a major impact on health and significant socioeconomic burden (1, 2). Overweight and obesity are often associated with a cluster of metabolic abnormalities, such as dyslipidemia, hypertension, and type 2 diabetes mellitus (T2D), which may lead to the development of metabolic syndrome syndrome (MetS) (3). In parallel with the growing obesity pandemic, the prevalence of T2D is also increasing worldwide, and it is expected to continue to rise in the coming years, resulting in devastating consequences (4). It is noteworthy that pancreatic beta cells are key players in the pathophysiology of T2D (5). Therefore, the central event in this condition consists of a relative insulin deficiency due to beta cell dysfunction, which often coexists with insulin resistance (5). In this regard, metabolic stress leads to beta cell apoptosis, which results in progressive loss of functional beta cell mass (5). Importantly, reciprocal interactions may occur among clustering components of MetS, leading to an increased risk for the development of cardiovascular disease (3). In line with this, central fat distribution related to MetS has been demonstrated to play a vital role in the pathophysiology of T2D, whereas disrupted glucose homeostasis and beta cell dysfunction may also promote visceral fat accumulation (6). However, some of the intricate connections and metabolic pathways involved in the crosstalk between adipose tissue and pancreatic beta cells remain poorly understood.

In recent years, the gut microbiome has emerged as a central player in the development, progression, and therapeutics of obesity and T2D (7). The human gut microbiota is composed of trillions of microorganisms located in the gastrointestinal tract that have a close symbiotic relationship with the host (8). Notably, bacterial metabolites, such as short-chain fatty acids (SCFAs), vitamins, amino acids, and bile acids (BAs), are also involved in essential bacteria and host cell-to-cell interactions (9). Therefore, when the fragile equilibrium between intestinal microbiota and host metabolism is disrupted, several disorders may develop, including overweight/obesity, ectopic fat accumulation, hyperlipidemia, insulin resistance, and hyperglycemia (10). Taken together, disturbed homeostasis between adipose tissue and pancreatic beta cells may be driven, in part, by pathological shifts in the gut microbiome and derived metabolites.

In this review, we discuss the main mechanisms involved in the interplay between adipose tissue and pancreatic beta cells, with special attention to the bidirectional influences leading to beta cell dysfunction/insulin resistance and adipocyte dysfunction. In addition, we summarize the novel insights into the role of the gut microbiome and related metabolites in the mediation of this complex crosstalk, including an integrative view of the relationship between adipose tissue-derived bacteria and beta cell/adipose tissue dysfunction.

## 2 Adipose Tissue and Pancreatic Beta Cell Communication: A Complex Dialogue

### 2.1 What Is the Role of Adipose Tissue in Beta Cell Dysfunction?

**Figure 1:**
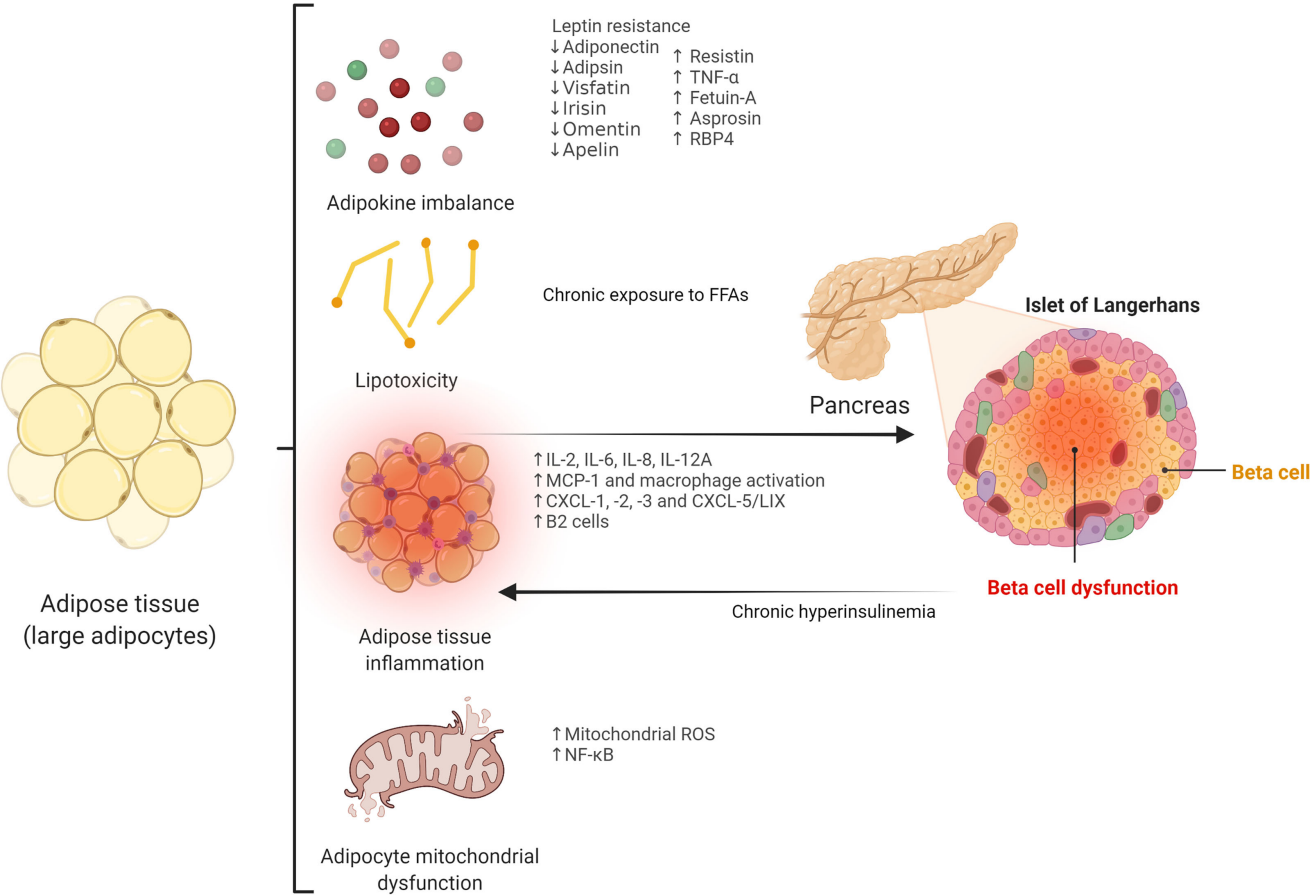
Potential adipose tissue-related mechanisms leading to beta cell dysfunction. TNF-α, tumor necrosis factor α; RBP4, retinol-binding protein 4; FFAs, free fatty acids; IL, interleukin; MCP-1, monocyte chemoattractant protein-1; CXCL, chemokine (C-X-C motif) ligand; CXCL-5/LIX, chemokine (C-X-C motif) ligand-5/lipopolysaccharide-induced CXC chemokine; ROS, reactive oxygen species; NF-κB, nuclear factor-kappa B.

Central distribution of adipose tissue, as opposed to peripheral locations (i.e., femoro-gluteal adipose tissue) is a well-known risk factor for the development of insulin resistance and T2D (11). Importantly, impaired subcutaneous adipose tissue expandability, determined by environmental and genetic factors, has been postulated as the main mechanism leading to visceral fat accumulation (12–14). Thus, when the adipose tissue storage capacity limit is reached, excess fat may accumulate in ectopic deposits, including key organs such as skeletal muscle, liver, and pancreas, constituting an important cause of insulin resistance and beta cell dysfunction (15). Beyond its storage function, adipose tissue is a metabolically active organ with a major role in beta cell dysfunction *via* different mechanisms, including adipokine production, lipotoxicity, and increased inflammatory response (**Figure 1**).

#### 2.1.1 Adipokines

Adipose tissue constitutes an important source of bioactive hormones, which are key factors in beta cell function and impairment. Among them, leptin and adiponectin have been extensively studied. Leptin exerts direct effects on pancreatic beta cells through the activation of the leptin receptor, which in turn stimulates the Janus-kinase (JAK)/signal transducer of activation (STAT) - mitogen-activated protein kinase (MAPK) signaling pathway (16). Leptin inhibits ectopic fat deposition in beta cells and reduces triglyceride accumulation in islets, preventing apoptosis and beta cell dysfunction, although its role in insulin secretion remains controversial (17, 18). Other mechanisms involved in apoptosis prevention by leptin include the inhibition of inducible nitric oxide synthase (iNOS) expression (19) and the regulation of B-cell lymphoma 2 (Bcl-2) and Bcl-2-associated X protein (Bax) (17). However, leptin may also exert harmful effects on beta cells. Thus, leptin increases the release of interleukin-1b (IL-1b) from beta cells and decreases the expression of the IL-1 receptor antagonist, leading to impaired beta cell function and apoptosis (20). Also, leptin has been reported to induce beta cell apoptosis and impairment of glucose-stimulated insulin secretion *via* c-Jun N-terminal kinase (JNK) activation (21). On the other hand, adiponectin has protective and anti-apoptotic effects on beta cells, and low levels of this adipokine have been associated with insulin resistance and beta cell dysfunction (22). Adipsin has also been reported to improve beta cell function, and its deficiency triggers beta cell failure and insulinopenia (23). Visfatin stimulates insulin secretion and inhibits beta cell apoptosis through the MAPK and phosphatidylinositol 3-kinase pathway (PI3K)/protein kinase B (AKT) pathway (24), whereas irisin improves glucolipotoxicity associated with beta cell dysfunction through adenosine monophosphate- activated protein kinase (AMPK) signaling and reduces the inflammatory response (25, 26). Decreased omentin levels may also be related to the development of T2D, since this adipokine has been demonstrated to have an influence on beta cell survival (27). Apelin significantly increased beta cell mass in preclinical models (28), although high concentrations of this adipokine were previously reported to inhibit insulin response to glucose (29). On the other hand, increased levels of some adipokines have been related to a negative impact on pancreatic beta cells. Thus, resistin induces insulin resistance and impairs insulin secretion in pancreatic beta cells *via* the increased expression of suppressor of cytokine signaling 3 (SOCS-3) and reduced AKT phosphorylation (30). In addition, tumor necrosis factor α (TNF*-* α), a pro-inflammatory cytokine and adipokine, induces beta cell apoptosis (31). Fetuin-A, a hepato-adipokine, leads to beta cell failure and apoptosis *via* the toll-like receptor-4 (TLR4)- JNK- nuclear factor-kappa B (NF-κB) signaling pathway (32). Recently, the novel adipokines asprosin and retinol-binding protein 4 (RBP4) have been reported as important players in the pathophysiology of T2D and beta cell dysfunction in preclinical studies. Thus, asprosin contributed to beta cell apoptosis by the inhibition of protective autophagy in beta cells through the AMPK-mammalian target of rapamycin (mTOR) pathway in *in vitro* models (33), whereas RBP4 has been shown to be stimulated by retinoic acid 6 (STRA6), which provoked pancreatic beta cell failure and T2D progression in rodent models (34).

#### 2.1.2 Lipotoxicity

Free fatty acids (FFAs) are released into the circulation from adipose tissue lipolysis, constituting an important energy source during starvation (35). Also, they are crucial signal transducing molecules in several pathways, including those involved in glucose metabolism, insulin resistance, and beta cell function (36). Despite the fact that the acute release of FFAs increases beta cell mass and insulin secretion (37), chronically elevated levels of FFAs inhibit glucose-stimulated insulin secretion and lead to beta cell dysfunction *via* cytotoxic mechanisms that result in beta cell apoptosis (38, 39). Thus, chronic exposure to FFAs is associated with ceramide synthesis, mitochondrial dysfunction, and overexpression of apoptotic genes in beta cells (40). Besides, FFAs trigger intracellular triglyceride accumulation in pancreatic beta cells promoted by the activation of sterol regulatory element-binding proteins (SREBPs) (41).

#### 2.1.3 Adipose Tissue Inflammation and Release of Pro-Inflammatory Factors

Visceral adipose tissue is able to secrete several pro-inflammatory factors, such as IL-2, IL-6, IL-8, IL-12A, or monocyte chemoattractant protein-1 (MCP-1), which may have a role in beta cell dysfunction (42, 43). Interestingly, recent data show that peripancreatic adipose tissue may have a strong influence on beta cell function, since close contact is established between this ectopic fat accumulation and islets of Langerhans, facilitating adipocyte-beta cell paracrine communication. Thus, increased expression of peripancreatic adipose tissue-derived factors, such as chemokine (C-X-C motif) ligand (CXCL)-1, -2, -3, and CXCL-5/lipopolysaccharide-induced CXC chemokine (LIX) acting on CXC receptor-2, as well as macrophage activation, have been shown to be implicated in the impairment of beta cell function (44). Moreover, additional organs may play a role in this equation: increased levels of hepatokine fetuin-A in non-alcoholic fatty liver disease induce impaired insulin secretion and islet cell death *via* the stimulation of peripancreatic adipocytes, which produce IL-6, IL-8, and MCP-1 through TLR4-dependent mechanisms (45).

Importantly, activated macrophages infiltrating adipose tissue are essential players in the development and maintenance of the pro-inflammatory state associated with harmful effects on pancreatic beta cells (46). Intriguingly, recent research has revealed that macrophages may also have an impact on beta cells independently of inflammatory mechanisms (i.e., *via* the release of miRNA-containing extracellular vesicles) (47, 48). Extracellular vesicles released by inflamed adipocytes can also cause beta cell death (49). Other adipose tissue-resident immune cells, such as B2 lymphocytes, may promote insulin resistance *via* the chemokine leukotriene B4 (LTB4) and its receptor, LTB4 receptor-1 (50).

Specific adipose tissue proteomic and transcriptomic profiles associated with inflammatory pathways may also be involved in beta cell dysfunction (51). Recently, the transcriptional coregulator GPS2 in white adipose tissue has been associated with beta cell insulin secretion (52).

Finally, adipocyte mitochondrial dysfunction and reactive oxygen species (ROS) overload may contribute to beta cell impairment. Thus, mitochondrial ROS pathway and NF-κB signaling have been associated with mitophagy-mediated adipose inflammation that promotes pancreatic beta cell damage (53).

### 2.2 What Is the Role of Beta Cells in Adipose Tissue Dysfunction?

Beta cells are key regulators of adipose tissue metabolism. Insulin exerts important anabolic effects on adipose tissue, including those involved in adipocyte function, growth, and differentiation (54). Insulin resistance and beta cell dysfunction are the two main mechanisms implicated in the pathogenesis of T2D, constituting a vicious cycle in which adaptive insulin hypersecretion to meet elevated metabolic demand is followed by the progressive loss of beta cell mass and function (55), and both conditions act synergistically in adipocyte dysfunction. In this line, chronic hyperinsulinemia has been reported to enhance adipose tissue inflammation and drive adipose tissue dysfunction in obese mice, and lowering circulating insulin levels was demonstrated to decrease macrophage content in adipose tissue (56). Hyperinsulinemia can also contribute to the pro-inflammatory M1:M2 macrophage imbalance in adipose tissue, which promotes iNOS, ultimately resulting in extracellular matrix deposition and adipose tissue fibrosis (57). Previous studies conducted in human subjects have revealed similar results. In this regard, Krogh-Madsen et al. found that hyperinsulinemia prompts IL-6 and TNF*-* α gene expression in adipose tissue (58). Of note, a recent study showed that chronic hyperinsulinemia leads to premature adipocyte senescence and a pro-inflammatory secretory profile *in vitro* and *in vivo* (59).

## 3 Gut Microbiome and Derived Metabolites, Additional Players in Beta Cell-Adipose Tissue Crosstalk

### 3.1 The Gut Microbiome Regulates Adipocyte and Beta Cell Function

Mounting evidence suggests that altered gut microbiome composition, known as gut dysbiosis, is involved in the development of adipose tissue dysfunction and insulin resistance/T2D (60). In line with this, gut barrier dysfunction and increased gut permeability, which results in the impairment of biological homeostasis by the translocation of bacterial toxins inducing systemic inflammation, may be a major factor related to these conditions (61). Thus, gut dysbiosis can affect the intestinal epithelial barrier by the modulation of the immune system, including TLR signaling, which regulates the integrity of tight junction complexes (61). Remarkably, some modulators of intracellular tight junctions and gut permeability, such as zonulin, may also play a crucial role (62). Accordingly, increased circulating levels of zonulin, an important marker of tight junction disassembly and increased gut permeability, have been correlated with gut dysbiosis and the development of metabolic disturbances (63–65). Apart from gut dysbiosis, additional factors, such as diet, should be taken into consideration in the pathogenesis of gut permeability and pro-inflammatory response in obesity and T2D (60).

With regard to the influence of the gut microbiome on adipose tissue, Bäckhed et al. reported for the first time that the gut microbiota was a key environmental factor in the predisposition towards adiposity, since it can regulate body fat storage and adipocyte metabolism (66). Indeed, the causative role of gut microbiota in the development of obesity is supported by mice models, which showed that an obese phenotype could be transferred through fecal microbiota transplantation (67, 68). Notably, a number of studies have revealed that some gut microbial patterns have a strong influence on adipose tissue inflammation, which constitutes one of the essential features in adipocyte dysfunction and may also lead to beta cell impairment, as previously described. In animal models, specific gut microbiota profiles have been demonstrated to drive Western-type diet-induced adipose tissue inflammation *via* myeloid differentiation primary response 88 (Myd88) and TLR signaling (69). Besides, increased intestinal permeability due to dysbiosis triggers the translocation of bacterial endotoxins that may have deleterious effects on adipose tissue. In line with this, intestinal permeability has been associated with increased visceral lipid deposition in healthy women (70). Also, elevated serum levels of lipopolysaccharide (LPS) from the Gram-negative bacterial membrane promote the inflammatory reaction in adipose tissue in obesity, including the pro-inflammatory activation of macrophages and adipocyte death by pyroptosis (71). Gut dysbiosis leads to the release of zonulin, which modulates immune response and increases gut permeability in distinct metabolic disorders, including obesity (64, 65). Of note, low serum levels of zonulin have been associated with high alpha diversity in pregnant women with obesity (72). Importantly, disruptions in the microbiome-immune-metabolic axis in early life, including gut barrier alterations and secondary immune-mediated inflammatory chronic activation related to childhood obesity, could impact adult overweight and obesity (73).

On the other hand, a growing body of evidence shows that the gut microbiome has a major role in the pathophysiology of T2D (74). Thus, bacterial genera such as *Ruminococcus*, *Fusobacterium*, and *Blautia* have been positively associated with this condition, whereas *Bifidobacterium*, *Bacteroides, Faecalibacterium, Akkermansia*, and *Roseburia* are inversely related to T2D (74). Moreover, increased gut permeability derived from gut dysbiosis may be related to the pathogenesis of T2D, as shown in preclinical studies (75). In this regard, higher zonulin levels have been reported in patients with a recent diagnosis of T2D, and may play a role in the pathophysiology of this disease, although further research is needed (76). Insulin sensitivity/resistance is also mediated by the gut microbiota (77). Interestingly, preclinical studies show that the loss of some beneficial bacteria, such as *Akkermansia muciniphila*, causes impaired intestinal integrity and systemic inflammation, leading to insulin resistance, while the increased abundance of this bacterium restores normal insulin response (78). Also, circulating levels of zonulin have been shown to be closely related to insulin resistance in clinical studies (64, 76). On the other side, clinical studies have revealed that calorie restriction may ameliorate insulin sensitivity through positive changes in the gut microbiota (79). Further research in humans has also corroborated that gut microbiota composition is closely linked to insulin resistance (80, 81). In addition, animal models have shown that gut microbiota is required for early beta cell development and proliferation (82), and gut microbiota signals (e.g., nucleotide-binding oligomerization domain-containing protein 1--NOD1-ligands derived from gut microbes) are needed for normal insulin biogenesis (83). In animal models showing that an obese phenotype can be transferred by fecal microbiota transplantation, mild glucose intolerance was an early manifestation in the host, a fact that suggests that the gut microbiome may affect both adipose tissue and beta cell function (68). Importantly, beta cell hyperactivity and subsequent hyperinsulinemia, which has a strong influence on adipose tissue dysfunction, can be transmitted early to recipient mice of obese microbiota despite only a minor increase in weight gain and adiposity (84). Also, hyperglycemia may increase gut permeability, which could aggravate metabolic inflammation and lead to the development of adipose tissue dysfunction and obesity (60).

**Figure 2:**
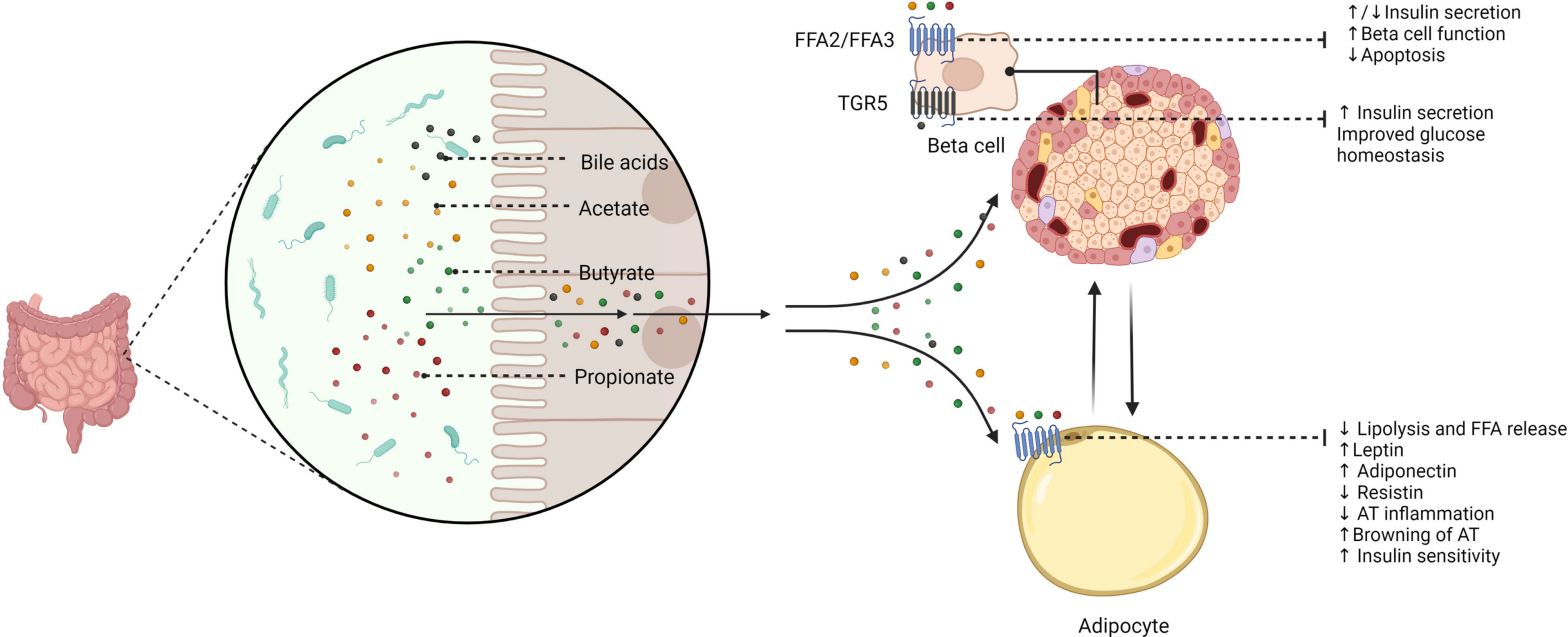
The potential role of gut microbiota-derived metabolites in beta cell and adipocyte function. The gut microbiome secretes several signaling molecules with direct effects on beta cell and adipocyte function. Short-chain fatty acids (SCFAs), including acetate, butyrate, and propionate, exert different effects on beta cells *via* binding short-chain fatty acid receptor-2 (FFA2) and FFA3. Thus, SCFAs inhibit apoptosis, improve beta cell function, and enhance insulin secretion. However, it has been reported that some SCFAs (i.e., acetate and propionate) could also inhibit insulin secretion. Bile acids may stimulate insulin secretion and improve glucose homeostasis through Takeda G-protein coupled receptor 5 (TGR5). SCFAs also have a role in adipocyte function *via* FFA2 and FFA3. Therefore, acetate, butyrate, and propionate regulate adipocyte metabolism and adipokine balance. These effects may result in reciprocal influences between beta cells and the adipocyte. FFA2/FFA3, short-chain fatty acid receptor 2/3; TGR5, Takeda G-protein coupled receptor 5; FFA, free fatty acids; AT, adipose tissue.

Remarkably, gut microbiota-related metabolites have direct effects on adipocyte and beta cell function (**Figure 2**). The gut microbiota secretes several molecules that reach key cells through specific receptors. By the fermentation of non-digestible dietary fibers, gut microbes produce SCFAs, including propionate, acetate, and butyrate, which exert direct actions through cell-surface G-protein-coupled receptors (GPCRs) (85). Additional bacterial products, such as amino acids, triglyceride metabolites, and BAs can also target these receptors (85). Pancreatic beta cells express SCFAs receptors-2 and 3 (FFA2/GPR43 and FFA3/GPR41), which have direct effects on insulin secretion; however, mixed results have been reported in this regard. On the one hand, acetate was proven to inhibit glucose-stimulated insulin secretion *via* FFA2 and FFA3 in mouse and human beta cells (86). Conversely, another study showed that acetate enhances glucose-stimulated insulin secretion through the activation of the parasympathetic nervous system, although these effects appear to be related to hyperphagia, ectopic lipid deposition, and insulin resistance (87). Further studies have confirmed that acetate stimulates insulin secretion (88, 89). Butyrate may prevent pro-inflammatory cytokine-beta cell dysfunction and induce insulin secretion (90, 91), whereas propionate improved beta cell function and insulin release in humans (92), although contrary results have also been described (93). Besides, transmembrane bile acid receptor Takeda G-protein coupled receptor 5 (TGR5) can enhance insulin secretion and improve glucose homeostasis (94, 95). FFA2 and FFA3 are also expressed by adipocytes and are mainly associated with the regulation of adipokine release and adipose tissue metabolism (96, 97). SCFAs may also induce the browning of adipose tissue (98). Interestingly, butyrate can modulate adipocyte expansion and favor adipogenesis and adiponectin production through the upregulation of peroxisome proliferator-activated receptor gamma (PPAR-γ) (99) and suppresses adipocyte inflammation *via* the inhibition of the NOD-like receptor family pyrin domain containing 3 (NLRP3) pathway (100). Similarly, propionate ameliorates adipose tissue inflammation (101), whilst acetate could lead to adipose tissue dysfunction by TNF*-*α-induced MCP-1 production (102).

### 3.2 Gut Microbiota: A Potential Link Between Adipose Tissue and Beta Cell Communication

In previous sections, we have discussed the role of lipotoxicity, adipose tissue inflammation, and altered adipokine expression in the development of beta cell dysfunction and insulin resistance. Since pathological shifts in gut microbiota composition and related metabolites may lead to adipose tissue dysfunction *via* the aforementioned mechanisms, derived consequences are expected in beta cell survival and function. Thus, *Faecalibacterium prausnitzii* decreases adipocyte inflammation and increases adiponectin expression in visceral adipose tissue, which is related to insulin-sensitizing effects (103). Similarly, *A. muciniphila* reverses adipose tissue inflammation and restores insulin sensitivity in T2D (104). In addition, *Akkermansia* has been shown to be an important predictor of serum levels of FFAs, which are involved in lipotoxicity and beta cell impairment, presenting an inverse relationship with them and the pro-inflammatory cytokine IL-6 (105). Notably, in a study evaluating the role of angiopoietin-like 4 (ANGPTL4) in metabolic dysfunction, the loss of the expression of this adipokine uncoupled visceral fat accumulation from glucose intolerance *via* the gut microbiota (106).

Gut microbiome-derived metabolites are also important intermediates of the adipose tissue-beta cell crosstalk. Tryptophan-derived compounds produced by the gut microbiota regulate miRNA-181 expression in white adipose tissue, involved in glucose tolerance and insulin sensitivity (107). Thus, a decrease in tryptophan-derived metabolites is associated with the overexpression of miRNA-181, which favors the development of adipose tissue inflammation, impaired glucose tolerance, and insulin resistance (107). It is also known that butyrate stimulates adipocyte differentiation and adiponectin expression, favoring insulin sensitivity (108), whereas propionate enhances leptin expression and reduces resistin expression, which are closely involved in beta cell function (109). On the other hand, gut microbiota metabolites modulate insulin sensitivity/resistance in the host, which in turn affects adipocyte function. Thus, elevated circulating levels of LPS in individuals with T2D activate TLR-2 expression and trigger immune response and inflammation in adipose tissue (110). Metabolic endotoxemia induced by LPS triggers insulin resistance and the subsequent expression of inflammatory markers in adipose tissue to a similar extent as a high-fat diet (111).

In light of the above, gut dysbiosis and impaired metabolite secretion appear to drive an altered adipokine balance and induce adipose tissue inflammation, a fact that ultimately results in insulin resistance and beta cell dysfunction, which can also aggravate adipocyte inflammation *via* the gut microbiota, perpetuating the vicious cycle. However, further mechanisms, such as the direct bacterial presence in adipose tissue, constituting a specific-tissue microbiota, have been postulated in this intricate relationship.

### 3.3 The Role of Adipose Tissue-Derived Bacteria in Adipocyte/Beta Cell Dysfunction

It is noteworthy that bacterial translocation from the intestine to adipose tissue due to increased gut permeability, as proposed by the “tissue microbiota hypothesis” (112), could have an impact on adipose tissue-beta cell crosstalk (113–117) (**Table 1**). Accordingly, in animal models, the presence of bacteria in adipose tissue was previously reported (118). In mice, a high-fat diet induced the translocation of Gram-negative bacteria through intestinal mucosa to circulation and mesenteric adipose tissue *via* pathogen-associated molecular patterns (PAMPs) recognition, Myd88 signaling, and leptin regulation, resulting in low-grade inflammation, linked to the early stages of T2D (113). Increased metabolic inflammation and insulin resistance have been associated with bacterial translocation from the intestine into adipose tissue in NOD2^-/-^ mice (114). Conversely, the identification of bacterial DNA in human adipose tissue has been a challenging task (119). Recently, the presence of specific microbial signatures in three different adipose tissues (omental, mesenteric, and subcutaneous adipose tissue) has been identified in subjects with morbid obesity, varying between individuals with and without T2D, with more evident signatures in mesenteric adipose tissue, including a decrease of health-promoting bacteria, such as *Faecalibacterium* and increased abundance of pathogens (e.g., *Enterobacteriaceae*) in subjects with T2D (115). In addition, Massier et al. also detected bacterial DNA in omental, mesenteric, and subcutaneous adipose tissue from 75 participants with obesity with or without T2D (116). Once more, mesenteric adipose tissue presented the highest bacterial quantity, which was associated with adipose tissue inflammation, and adipose tissue microbiota composition was different between subjects with and without diabetes (116). However, devoted clinical studies are needed to confirm these results.

**Table 1:** Animal models and clinical studies assessing the potential association between adipose tissue-derived bacteria and adipose tissue function/glucose homeostasis.

Study	Animals/Participants	Adipose tissue bacteria	Adipose tissue-related findings	Glucose homeostasis-related findings
Amar et al. (113)	NC/HFD-fed mice	Gram-negative bacteria (experimental translocation model).	Increased TNF*-α* and IFN-γ in MAT, correlating with bacterial DNA concentration.	Increasing MAT bacterial DNA concentration in the progression of prediabetes to diabetes. Probiotic treatment reduced mucosal dysbiosis, bacterial translocation, and improved glucose metabolism.
Denou et al. (114)	NOD2^-/-^ mice	Commensal bacteria (experimental translocation model).	Increased inflammation (IL-6, TNF*-α*) in visceral adipose tissue.	Increased insulin resistance.
Ahnê et al. (115)	Subjects with morbid obesity with T2D (n-20) and without T2D (n-20)	Different compartmentalization according to specific tissue (MAT, OAT, SAT).	Not assessed.	More evident T2D signatures in MAT: reduced bacterial diversity and Gram-positive bacteria (i.e., *Faecalibacterium*) and increased Gram-negative *Enterobacteriaceae*.
Massier et al. (116)	Subjects with obesity with T2D (n-33) and without T2D (n-42)	*Proteobacteria* and *Firmicutes* were the predominant phyla in adipose tissue (MAT, OAT, SAT). Higher bacterial quantity and diversity in MAT.	Bacterial DNA correlated with macrophage infiltration in OAT (especially in T2D), TNF*-α* in SAT, and IL-1B in MAT; bacterial DNA induced adipokine secretion.	Eighteen genera were shown to present different abundance between subjects with T2D and subjects without T2D.
Bakker et al. (117)	Subjects with obesity and metabolic syndrome receiving lean donor FMT (n-8); BMI- matched controls not receiving FMT (n-16)	Very low quantity of bacterial DNA in visceral adipose tissue.	FMT did not alter bacterial translocation to adipose tissue. No differences in visceral bacterial DNA content/macrophage infiltration between groups.	Not assessed.

NC, normal chow; HFD, high-fat diet; MAT, mesenteric adipose tissue; OAT, omental adipose tissue; SAT, subcutaneous adipose tissue; TNF- α, tumor necrosis factor α; IFN- γ, interferon γ; NOD2, oligomerization domain-2; IL-6, interleukin 6; IL-1B, interleukin-1B; TD2, type 2 diabetes mellitus; FMT, fecal microbiota transplantation; BMI, body mass index.

### 3.4 Impact of Gut Microbiome Modulation on Adipose Tissue-Beta Cell Crosstalk

The gut microbiome may be targeted to modulate the metabolic dialogue between adipose tissue and pancreatic beta cells. Hence, prebiotic approaches [i.e., non-digestible food components that benefit the host by the selective stimulation of the growth/activity of specific bacterial strains (120)] have emerged as promising interventions. Oligofructose supplementation in high-fat diet-fed mice increased gut *Bifidobacterium* spp. and prevented the elevation of adipose tissue inflammatory markers, which was linked to the improvement of glucose tolerance and the restoration of glucose-induced insulin secretion (121). Moreover, an oligofructose-enriched diet decreased *Firmicutes* and increased *Bacteroidetes* abundance, reducing adipose lipid peroxidation and ameliorating leptin sensitivity and glucose tolerance (122). The combination of the dietary flavonoid isoquercetin with soluble fiber (inulin) attenuated weight gain, improved glucose tolerance/insulin sensitivity, reduced adipocyte hypertrophy/ectopic fat accumulation, and restored adipokine balance in high fat diet-fed mice (123). On the other hand, the direct administration of health-promoting live microorganisms (probiotics) could confer several benefits. Lactic acid bacteria strains were demonstrated to modulate the adipokine profile in *in vitro* models (124). Besides, probiotic interventions targeting key gut microbes in the protection against adipocyte/beta cell dysfunction, such as *A.muciniphila* and *F.prausnitzii*, may constitute an attractive approach (103, 104, 125). Postbiotics, defined as bioactive substances produced by microorganisms with positive effects on the host (126), can also modulate adipocyte and beta cell function. The previously discussed SCFAs are relevant postbiotics in this regard (85, 108, 109). The combination of inulin and SCFAs reduced adipocyte size and prevented diet-induced obesity and insulin resistance in animal models (127). Interestingly, the administration of the natural metabolite 4-cresol reduced adiposity and enhanced insulin secretion and beta cell proliferation in mouse islets (128). Fecal microbiota transplantation from lean donors to patients with obesity and metabolic syndrome transiently improved insulin sensitivity (129), and animal models have revealed that this therapy may reverse beta cell dysfunction (130). However, further research is needed to confirm these results.

## 4 Concluding Remarks

Obesity and T2D are increasing in prevalence, resulting in major health and socioeconomic consequences. The relationships between these two disorders are well established; however, some of the underlying mechanisms involved in their pathophysiology and bidirectional links are not fully understood. Pancreatic beta cells and adipose tissue are closely interconnected through the presence of a number of bioactive hormones and intricate signaling pathways. Also, the gut microbiome may play a key role in the mediation of the complex dialogue between the adipocyte and beta cell, with derived potential therapeutic strategies in this field. However, important issues are yet to be elucidated. Cells do not live in isolation, and multiple interactions are expected to occur beyond the dialogue among the gut microbiome, adipose tissue, and pancreatic beta cells. Therefore, additional players, such as the skeletal muscle and the liver, may be included in this metabolic crosstalk. Future perspectives in this area should also focus on the development of therapeutic approaches (e.g., nutritional therapy) targeting the gut microbiota and the distinct dysfunctional metabolic pathways. Finally, dedicated clinical studies are warranted to fully unravel the role of the gut microbiome and related metabolites in the crosstalk between pancreatic beta cells and adipose tissue.

## Author Contributions

Conceptualization, JM-M and FT. Investigation, JM-M, MD-F, and JF-G. Original draft preparation, JM-M and MD-F. Writing- review and editing, JM-M, JF-G, and FT. Supervision, FT. All authors contributed to the article and approved the submitted version.

## Funding

MD-F was supported by Rio Hortega from the Spanish Ministry of Economy and Competitiveness (ISCIII) and co-funded by Fondo Europeo de Desarrollo Regional-FEDER (CM20/00183). JF-G was supported by an intensification research program (INT21/00078, ISCIII, Spain; co-funded by the Fondo Europeo de Desarrollo Regional-FEDER). This study was supported by the “Centros de Investigación Biomédica en Red” (CIBER) of the Institute of Health Carlos III (ISCIII) (CB06/03/0018), and research grants from the ISCIII (PI18/01160), and co-financed by the European Regional Development Fund (ERDF).

## Conflict of Interest

The authors declare that the research was conducted in the absence of any commercial or financial relationships that could be construed as a potential conflict of interest.

## Publisher’s Note

All claims expressed in this article are solely those of the authors and do not necessarily represent those of their affiliated organizations, or those of the publisher, the editors and the reviewers. Any product that may be evaluated in this article, or claim that may be made by its manufacturer, is not guaranteed or endorsed by the publisher.
